# Protease-Activation of Fc-Masked Therapeutic Antibodies to Alleviate Off-Tumor Cytotoxicity

**DOI:** 10.3389/fimmu.2021.715719

**Published:** 2021-08-03

**Authors:** Adrian Elter, Desislava Yanakieva, David Fiebig, Kerstin Hallstein, Stefan Becker, Ulrich Betz, Harald Kolmar

**Affiliations:** ^1^Institute for Organic Chemistry and Biochemistry, Technical University of Darmstadt, Darmstadt, Germany; ^2^Protein Engineering and Antibody Technologies, Merck Healthcare KGaA, Darmstadt, Germany

**Keywords:** Fc gamma receptor, off-target cytotoxicity, effector function, Fc-silencing, masked therapeutic antibody, MMP-9, ADCC, CDC

## Abstract

The interaction of the Fc region of therapeutic antibodies and antibody-drug conjugates with Fcγ receptors (FcγRs) can lead to unpredictable and severe side effects. Over the last decades several strategies have been developed to overcome this drawback, including extensive Fc- and glycoengineering and antibody isotype switching. However, these approaches result in permanently Fc-silenced antibody derivates which partially or completely lack antibody-mediated effector functions. Nevertheless, for a majority of antibody-based drugs, Fc-mediated effector functions, like antibody-dependent cell-mediated cytotoxicity (ADCC), antibody-dependent cell-mediated phagocytosis (ADCP) as well as complement-dependent cytotoxicity (CDC), represent the most substantial modes of action. We argued that a new strategy combining the beneficial properties of Fc-silencing and controlled activation of effector functions can pave the way to potent antibody therapeutics, reducing the FcγRs-mediated off-target toxicity. We present a novel Fc-tamed antibody format, where the FcγR-binding sites of antibodies are blocked by anti-isotypic masking units, hindering the association of FcγR and complement component 1 (c1q) to the Fc domain. The masking units were genetically fused to trastuzumab, including a protease-addressable peptide-liker. Our Fc-tamed antibodies demonstrated completely abolished interaction to soluble high-affinity Fcγ-Receptor I and c1q. In reporter cell-based ADCC assays, our Fc-tamed antibodies exhibited a 2,700 to 7,100-fold reduction in activation, compared to trastuzumab. Upon demasking by a tumor-associated protease, the Fc-activated antibodies demonstrated restored FcγR-binding, c1q-binding and the ability to induce potent ADCC activation. Furthermore, cell killing assays using donor-derived NK cells were performed to validate the functionality of the Fc-tamed antibody variants. To our knowledge, this approach represents the first non-permanently Fc-silenced antibody, which can be re-activated by a tumor-associated protease, eventually extending the field of novel antibody formats.

## Introduction

**Figure 1:**
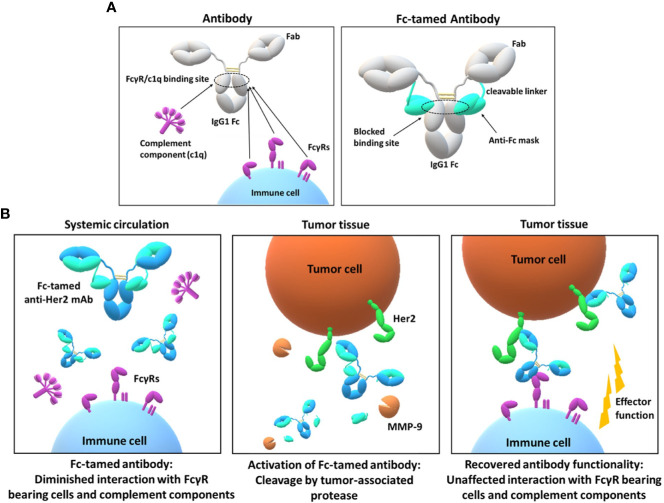
Design and mode of action of the Fc-tamed antibodies. **(A)** Depiction of overlapping FcγR and c1q interaction sites located on the CH2 domain of an IgG1 antibody (left panel). Molecule design of the Fc-tamed antibodies, comprising two antibody heavy chains (IgG1) and two light chains, C-terminally fused to a protease-cleavable linker and a FcγR-blocking anti-isotypic masking unit (right panel). **(B)** Conceptional mode of action of a Fc-tamed anti-Her2 monoclonal antibody. After administration and during systemic circulation, Fc-tamed antibodies exhibit diminished interaction to FcγR-bearing cells and complement components (c1q) (left panel). Entering the tumor microenvironment, the Fc-tamed antibodies are activated upon linker proteolysis by tumor-specific proteases (MMP-9) (middle panel). The active antibody demonstrates recovered ability to induce antibody-mediated effector functions (right panel).

In the last decades monoclonal antibodies (mAbs) became powerful and promising drug classes, due to their ability to selectively address cancer-related molecules, infectious cells, virus particles, immune cells and immune-checkpoint-related molecules. As a part of the immunoglobulin isotype family, the immunoglobulin G (IgG) class, particularly the IgG1 subclass ranks as the most dominant isotype used for therapeutic applications (1). IgGs can induce cell-mediated (ADCC, ADCP) and complement-mediated (CDC) effector functions by interacting with Fcγ receptors (FcγRs) on immune cells or complement components, present in serum. Thereby, the different IgG subclasses (IgG1, IgG2, IgG3, IgG4) display unique FcγR and complement component binding profiles (2). All FcγRs (FcγRI, FcγRIIa, FcγRIIb, FcγRIIc, FcγRIIIa, and FcγRIIIb) address similar epitope regions, located in the lower hinge/upper C_H_2 region of antibody Fc, including the N297-linked glycan structure (3). While the FcγRI is able to bind to monomeric IgG with low nanomolar affinity, all other FcγRs display high nanomolar to low micromolar equilibrium dissociation constants (K_D_) and thus, predominantly bind to immune complexes (3). The affinity of FcγRs and, consequently, the versatile downstream signaling differ, depending on the antibody isotype and antibody glycosylation. Additionally, polymorphisms of FcγRs show an immense influence on the affinity to different subclasses of IgGs, translating in reduced or enhanced efficacy of therapeutic antibodies (4, 5). FcγRs are expressed by the majority of white blood cells, including monocytes, macrophages, dendritic cells, mast cells, B cells, NK cells, all comprising a different FcγR expression profile (6–8). Antibody-dependent cell-mediated phagocytosis, antibody-dependent cell-mediated cytotoxicity and complement-dependent cytotoxicity contribute to the most important modes of action of currently approved antibody therapeutics. However, several adverse side effects, including uncontrolled cytokine release, myelosuppression, blood platelet aggregation, thrombocytopenia and allodynia are linked to unwanted Fc-FcγR ligation or complement activation (9–11). Multiple strategies for the prevention of undesired Fc-FcγR interaction have been developed over the last decades (12, 13). Most approaches account to the implementation of several point mutations within the FcγR interaction site or deglycosylation in position N297, leading to a partial or complete decline of FcγR binding. In case of an anti-CD3 monoclonal antibody, two amino acid substitutions (L234A, L235A) resulted in reduction of severe side effects (14). Furthermore, several studies reported a correlation of Fc receptor binding-related internalization of antibodies and antibody-drug conjugates (ADCs) and adverse side effects (e.g. thrombocytopenia) (10, 15–17). To circumvent thrombocytopenia upon administration of ADCs several point mutations can be introduced to minimize FcγR binding (18). A prominent example is the implementation of three single point mutations in the Fc part of an anti-HER2 tubulysin (IgG1) ADC (results from clinical trial phase 1) in order to reduce FcγR-related side effects (19). Similarly, a single point mutation (K322A) is known to limit the interaction of C1q to the IgG1 Fc domain, which resulted in reduced antibody-induced allodynia *in vivo* (20). Although several Fc-engineered antibodies have been approved for clinical use, all approaches result in permanently silenced and structurally altered Fc domains. In recent years, research focused on masking the paratopes of antibodies to ensure the selective activation of antibody binding properties (21, 22). This technology requires the generation of a suitable masking unit preventing antibody-antigen binding either by a steric hindrance or due to a specific interaction with the antibody paratopes (23). Demasking and consequently activation of the antibody is typically mediated by proteases, which are upregulated in malignant tissues and potentially serve as diagnostic marker molecules (24, 25). Previous masking strategies resulted in antibody therapeutics with improved safety, while maintaining anti-disease activity in clinical trials (26, 27). However, all reported approaches exclusively addressed the antigen binding fragments (paratopes) of antibodies, overlooking the interaction sites for FcγRs and complement components on the Fc part. Inspired by these studies, we reasoned that a temporarily Fc-masked antibody combined with selective demasking by a tumor-associated protease can further extend the benefit of Fc-silencing technologies. In our concept, the antibody Fc domain remains inert during circulation and restores effector function properties upon reaching the malignant tissue, thereby potentially widening the therapeutic window and improving the efficacy of the antibody drugs. In this study, we developed an Fc-tamed antibody format, starting with the generation of an anti-isotypic masking unit, originating from a chicken-derived (humanized) single-chain variable fragment (scFv), which specifically binds the interaction site of FcγRs on the IgG1 Fc. The final masking unit, comprising the scFv and a matrix metallopeptidase 9 (MMP-9)-addressable linker was genetically fused to the C-terminus of the light chain of an anti-Her2 antibody (trastuzumab) (**Figure 1A**). The resulting Fc-tamed anti-Her2 antibody demonstrated substantially reduced FcγR- and c1q-interaction properties. Upon MMP-9-mediated cleavage of the linker and dissociation of the masking unit, the Fc-activated antibody displayed fully recovered binding to FcγR and c1q, as well as restored antibody-dependent effector functions (**Figure 1B**).

## Material and Methods

### Chicken Immunization and Yeast Surface Display

Chicken immunization, the construction of a scFv yeast surface display library, yeast cell cultivation and scFv-display induction were performed as described previously (28, 29). Briefly, an adult chicken was immunized with a Fc-fusion protein. For library construction, cDNA synthesis of total spleen cell RNA and the amplification of VH and VL coding genes enabled the transfer into a YSD vector (pCT) *via* transformation of yeast cells (Saccharomyces cerevisiae EBY100). EBY100 cells were incubated and induced using SD-CAA or SG-CAA, respectively.

### Fluorescence-Activated Cell Sorting and Flow-Cytometry

After incubation in (SG-CAA medium, 30°C, 180 rpm) yeast cells were separated using centrifugation. Cells were washed with 1ml PBS-B (137 mM NaCl, 2.7 mM KCl, 10 mM Na_2_HPO_4_, 1.8 mM KH_2_PO_4_, 0.1% (w/v) BSA, pH 7.4). Detection of scFv surface presentation, target binding (Fc-binding) and simultaneous FcγR-binding was enabled using primary and secondary labeling agents listed in **Table S1** (SI Appendix, **Table S1**). Staining procedure was performed according to **Table S2** (SI Appendix, **Table S2**). Conjugation of proteins to DyLight650 (Thermo Fisher) was performed using 5-fold excess of NHS-DyLight650. All centrifugation, wash and incubation steps were performed in PBS-B at 4°C. Cell sorting was performed using BD Influx cell sorter and the BD FACS software 1.0.0.650. Yeast cells isolated *via* FACS were cultivated in SD-CAA medium or on SD-CAA-agar plates. In order to evaluate Her2-binding of antibody variants, Her2-positive SK-BR-3 cells were trypsinized and washed with PBS-B followed by incubation in 50 µl of antibody solution for 30 minutes (5 × 10^5^ cells/well). Cells were washed two times with 200 µl PBS-B, followed by 30 minutes incubation in 50 µl of Goat anti-Human Kappa PE (SouthernBiotech) (1:75 diluted in PBS-B). Finally, cells were washed three times with 200 µl PBS-B and subsequently analyzed using CytoFLEX V3-B4-R3 Flow Cytometer (Beckman Coulter). The mean fluorescence intensity was plotted against the respective antibody concentration. Error bars represent standard deviation derived from experimental duplicates.

### Antibody Humanization

The humanization of a chicken-derived scFv was performed as described previously (30). Briefly, all six CDRs of scFv Fc4 were grafted onto human germline acceptor VH and VL frameworks (VH3-25/JH4, VL3-m/JL2). Partial randomization of Vernier residues was performed. A yeast surface display library with humanized scFv variants of scFv Fc4 was generated and screened *via* FACS for Fc-binding scFvs. The most abundant single clone was expressed and used for further experiments (**Figure S4**).

### Protein Expression and Purification

Vector reformatting, protein expression and purification of scFvs was performed as described previously (28). Briefly, scFv coding genes were reformatted into a pET30 vector using golden gate cloning and recombinant expression in *E. coli* T7 Shuffle express was performed. A 2-step purification using IMAC and Streptactin purification was performed, followed by buffer exchange against PBS pH 7.4 (137 mM NaCl, 2.7 mM KCl, 10 mM Na_2_HPO_4_, 1.8 mM KH_2_PO_4_). Protein expression of TRZ-hFc4, TRZ-Fc4 and TRZ-ctrl was performed in Expi293F cells. Prior, a CMV-controlled expression vector was ordered (TwistBioscience), exhibiting the light chain coding sequence of trastuzumab fused to the newly designed 33 amino acid linker coding sequence (**Figure S5**) followed by a scFv insertion site and a C-terminal StrepII tag. ScFv coding genes were inserted using golden gate cloning. A pTT5-derived CMV-controlled expression vector coding for the heavy chain of trastuzumab was used. ExpiFectamine 293 Transfection Kit (Gibco Biosciences) was used for transient transfection of Expi293F cells according to manufacturer instructions. After 5 days, cell culture supernatants were applied to a HiTrap KappaSelect 1-ml column using an Äkta Pure 25L FPLC system. Buffer exchange against TBS pH 7.4 (50 mM Tris-HCl, 150 mM NaCl) was performed using a 5-ml HiTrap Desalting column (GE Healthcare). Protein purity was confirmed *via* SDS-PAGE analysis using 4-15% Mini-PROTEAN protein gels (Biorad).

### Cell Lines

SK-BR-3 and Daudi cells were cultivated at 37°C and 5% CO_2_. Expi293F cells were cultivated at 37°C and 8% CO_2_. Prior to flow-cytometric analysis SK-BR-3 cells were detached using trypsin and washed with PBS-B. Several days before using Daudi cells in an ADCC reporter assay, cells were conditioned using Low IgG Serum (G711A, Promega).

### Matrix Metalloproteinase 9 Protein Hydrolysis

Recombinant active human MMP-9 (Sigma Aldrich) was used to cleave the MMP-9 cleavable light chain linker of TRZ-hFc4 or TRZ-Fc4. Proteins were dissolved in TBS pH 7.4, ensuring MMP-9 compatible conditions. 500 µl (0.25 mg/ml) of the respective antibody variant was mixed with 0.2 µl (1 mg/ml) of active human MMP-9. Protein hydrolysis was performed at 37°C for 48 h. Complete linker hydrolysis was confirmed using SEC and SDS-PAGE under non reducing conditions.

### Bio-Layer Interferometry

For affinity determination and epitope binning the Octet RED96 system (ForteBio, Molecular Devices) was used. For epitope binning of scFv Fc4, anti-human Fab-CH1 Biosensors (Fab2G) (ForteBio, Molecular Devices) were soaked in PBS pH 7.4 for at least 10 minutes. Cetuximab (20 µg/ml) was loaded to the sensor tip followed by a quenching step using Kinetics Buffer (KB) (ForteBio) diluted 1:10 in PBS pH 7.4. A first association step of 100 nM of scFv Fc4 followed by a second association step using 100 nM His-tagged human Fc gamma RI (CD64) His-tagged (Acro Biosystems) was performed. For each association step a negative control (only KB) was conducted. For affinity determination of scFv Fc4 and scFv hFc4 anti-human Fab-CH1 Biosensors (Fab2G) (ForteBio, Molecular Devices) were used to immobilize cetuximab (20 µg/ml). After a quenching step in KB, an association step using scFv Fc4 with concentrations ranging from 6.25 to 50 nM was performed followed by a dissociation step in KB. For affinity determination of scFv hFc4 a similar protocol was performed using scFv hFc4 concentrations ranging from 25 to 200 nM. Epitope binning of TRZ-scFv fusion proteins was performed using High Precision Streptavidin (SAX) Biosensors (ForteBio, Molecular Devices). Biotinylated Her2 (Sinobiological) was loaded on sensor tips followed by a quenching step with 100 ng/ml Biocytin (Thermo Fisher Scientific) and a second quenching step using KB. A first association step using 100 nM of the respective TRZ-scFv fusion protein or trastuzumab (Roche) was followed by a second association using 100 nM His-tagged human Fc gamma RI (CD64) (Acro Biosystems). Affinity determination of TRZ-scFv variants to FcγRI or human complement C1q (Sigma Aldrich) was performed using anti-human Fab-CH1 Biosensors (Fab2G). Association to different concentrations of FcγRI (6.25, 12.5, 25, 50 nM) was followed by a dissociation step in KB. A similar procedure was performed in case of c1q affinity determination using c1q concentrations ranging from 12.5 to 100 nM (12.5, 25, 50, 100 nM). In order to evaluate, whether the masking units of TRZ-hFc4 and TRZ-Fc4 interact with other Fc domains, biotinylated Her2 was loaded onto SAX sensor tips, followed by a first association step of 100 nM of TRZ-hFc4 or TRZ-Fc4. After first association, a second association step using 500 nM Cetuximab was performed. All proteins used in BLI experiments were diluted in Kinetics buffer (KB). Control samples (only KB) were subtracted prior to analysis. All kinetic calculations are based on at least 4 different protein concentrations. Data analysis was performed using ForteBio data analysis software 9.0. Binding kinetics were determined using Savitzky-Golay filtering and 1:1 Langmuir modeling.

### Size Exclusion Chromatography

Analytical size exclusion chromatography was performed using the Agilent Technologies 1260 Infinity device and a TSKgel SuperSW3000 column (Tosoh). 8 µg of protein was injected with a constant flow rate of 0.35 ml/min using TBS pH 7.4.

### Nano Differential Scanning Fluorimetry

Thermal stability of proteins was determined using Prometheus NT.48 device (NanoTemper Technologies). Briefly, 10 µl of protein solution (0.25 mg/ml in TBS) was applied to NT.48 Grade High Sensitivity Capillaries (NanoTemper Technologies). A temperature gradient of 1°C/min was applied. Analysis of fluorescence signal at 330 nm as well as the following calculation of thermal stabilities was performed using PR.ThermControl software.

### Antibody-Dependent Cell-Mediated Cytotoxicity

For ADCC activity determination, Promega ADCC Reporter Bioassay Kit (G7010) (Promega) was used. Assays were performed according to manufacturer instructions. A serial dilution (1:10) of rituximab (Roche) ranging from 6.6 fM to 66 nM was applied to CD20-positive Daudi cells. The same serial dilution was applied using Rituximab mixed with 10 molar equivalents of scFv Fc4. Her2 positive SK-BR-3 cells were seeded in 96-well plates and incubated for 24 h before use. Serial dilutions (1:10) of Trastuzumab, as well as TRZ-hFc4, TRZ-Fc4, TRZ-ctrl and MMP-9 treated TRZ-hFc4 and TRZ-Fc4 ranging from 10 fM to 100 nM were prepared. Per well, 10,000 target cells and 150,000 effector cells were co-incubated for 6 h at 37°C and 5% CO_2_. Luminescence intensity was measured and the fold induction (relative to control sample without any antibody) was plotted against the antibody concentration. Error bars represent standard deviations derived from experimental duplicates. For NK cell based ADCC assays, whole blood samples of healthy human donors were used to isolate PBMCs *via* gradient centrifugation. The ADDC assays were performed according to Pekar et al. (31). EasySep Human NK isolation Kit was used to isolate NK cells. NK cells were incubated overnight in complete medium (AIM V) supplemented with 100 U/ml recombinant human IL-2 (R&D Systems, Cat: 10453-IL). Target cells (SK-BR-3 or HCC-1954) were stained with CellTrackerDeep Red Dye (Thermo Fisher Scientific, Cat: C34565) according to the manufacturer’s protocol. 12,500 NK cells were mixed with 2,500 target cells and 5 µl of the respective antibody dilution in a 384-well clear bottom microtiter plate (total volume of 45 µl/well). Dead cell staining was achieved using SYTOX Green Dead Cell Stain (Invitrogen) at a final concentration of 30 nM. Antibody-mediated NK cell independent cytotoxicity was determined by incubation of target cells with the highest antibody concentration. Basal killing was determined incubating NK cells with target cells without the addition of antibody. For normalization, maximal cell killing was achieved by incubation with 0.25% Triton X-100. Cells were incubated at 37°C and 5% CO_2_. In case of time resolved ADCC assays, every 60 minutes cells were imaged for using Incucyte Live Cell Analysis System (Sartorius). The ratio of dead target cells to all target cells was used to determine antibody-dependent NK cell-mediated killing.

## Results

### Generation of FcγR-Blocking Anti-Isotypic Masking Units

**Figure 2:**
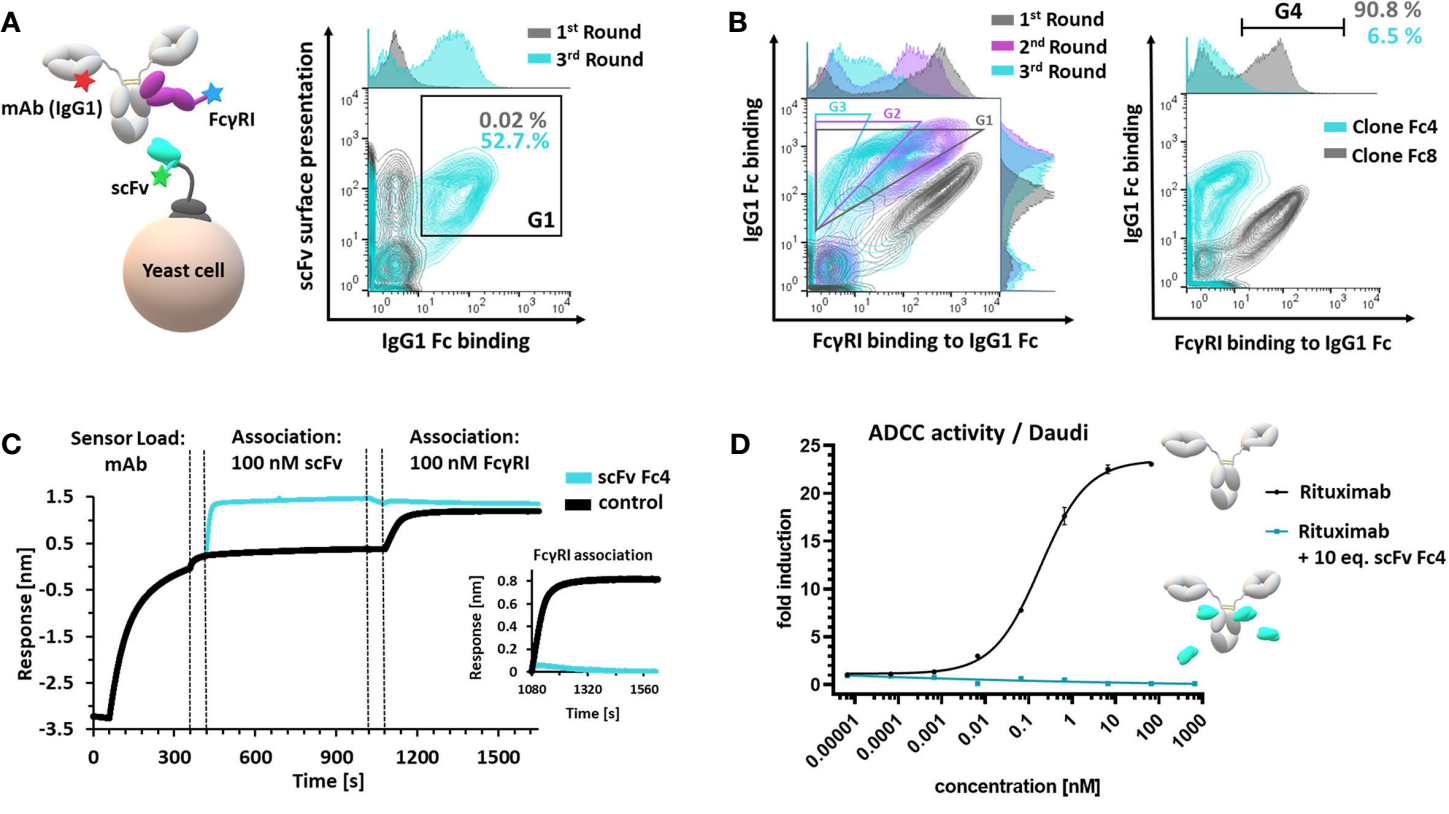
Generation and characterization of a FcγR-blocking anti-isotypic masking unit. **(A)** Schematic depiction of scFv yeast surface display *via* scFv-aga2p-aga1p system. scFv display (c-terminal c-myc tag), antibody (mAb(IgG1)) and simultaneous FcγRI binding was verified using fluorophore-conjugated proteins and fluorophore-conjugated detection reagents (left panel). Enrichment of Fc-binding scFvs – comparison of the first and third sorting round. Cells, displaying a functional c-myc tag and demonstrating Fc-binding (G1) were sorted in three consecutive FACS rounds (right panel). **(B)** Overlay of three consecutive FACS rounds, sorting for scFv-displaying cells binding to Fc and simultaneous blocking the FcγRI interaction (first round: G1; second round: G2; third round: G3) (left panel). Single clone analysis of single clones Fc4 and Fc8. Cells located in G4 binding to Fc and simultaneous displaying FcγRI binding. **(C)** BLI epitope binning. Monoclonal antibody (cetuximab, IgG1) was loaded onto Fab2G sensor tips. Control sample comprised no scFv during the first association step. Dashed lines represent 60 seconds- quenching steps using kinetics buffer. **(D)** ADCC reporter-cell assay using CD20-positive Daudi cells. Rituximab (IgG1) alone or rituximab (IgG1) pre-incubated with 10 molar equivalents scFv Fc4 was used. Error bars represent standard deviation derived from experimental duplicates.

For the generation of a suitable masking unit, which specifically targets the FcγR-binding site of antibodies, we focused on implementation of anti-isotypic (anti-IgG1) single-chain variable fragments (scFvs). The FcγR interaction sites located in the C_H_2 domain of IgGs are highly conserved in mammals (3), thus the probability of obtaining specific binders by immunization of mammalian species (mouse, rabbit, goat etc.) is considerably low. Nevertheless, previous studies demonstrated that immunization of chickens can provide access to antibodies targeting epitopes, which are conserved throughout mammalian species (32, 33). Hence, we reasoned that chicken immunization could facilitate the generation of scFvs targeting the FcγR interaction site on the human IgG1-Fc. Furthermore, chicken-derived antibodies can easily be humanized using established technologies (30, 33). An adult egg-laying hen (*Gallus gallus domesticus*) was immunized with an IgG1 Fc fusion-protein (28). Using scFv-yeast surface display (**Figure 2A**) and fluorescence-activated cell sorting (FACS) we could enrich Fc-binding scFvs (**Figure 2A**, G1) within three rounds of sorting (**Figures 2A** and **S1**). Three additional FACS rounds were conducted, gating for scFv-displaying cells which bind to IgG1 mAbs and simultaneously block the Fc-FcγRI interaction (34) (**Figure 2B**, G1, G2, G3, **Figure S2A**) enabling the isolation of 3 unique FcγRI-blocking scFv-displaying yeast clones (Fc1, Fc3, Fc4) (**Figures 2B** and **S2B, C**). Affine binding of a single clone Fc4 to 500 nM IgG1-Fc was verified by flow cytometry, while only neglectable binding (6.5%) to soluble FcγRI was observed. In contrast, an FcγRI-non blocking scFv clone, Fc8 displayed similar binding to IgG1-Fc, compared to Fc4 while 90.8% of Fc-binding cells demonstrated simultaneous binding to FcγRI (**Figure 2B**, G4). scFv Fc4 was heterologously expressed in *E. coli*, purified *via* IMAC (**Figure S3A**) and its affinity towards the Fc of a monoclonal antibody (cetuximab) was determined using Bio-layer interferometry (BLI). The scFv Fc4 exhibits an equilibrium dissociation constant (K_D_) of 2.3 nM (**Figure S3C**). Epitope binning for verification of FcγRI-blocking was also performed using BLI. Upon sensor tip loading using a monoclonal IgG1 antibody (cetuximab) and following association of scFv Fc4, no association of 100 nM soluble FcγRI could be detected, while cetuximab without prior scFv association demonstrated expected binding towards FcγRI, confirming specific binding of scFv Fc4 within the interaction site of FcγRs (**Figure 2C**). To investigate the blocking of further Fcγ receptor family members by scFv Fc4, a reporter cell-based ADCC assay was performed. The ADCC reporter cell assay is based on an engineered effector cell line stably expressing the high-affinity variant of FcγRIIIa (FcγRIIIa V158). In combination with CD20-positive target cells (Daudi), anti-CD20 mAb (rituximab) induced potent ADCC activation, while incubation of the target and effector cells with rituximab mixed with 10 molar equivalents of scFv Fc4 completely abolished the reporter gene expression over a wide range of antibody concentrations (**Figure 2D**). This indicates that the scFv Fc4 efficiently blocks the antibody-mediated cell-cell interaction. scFv humanization of Fc4 was performed using an established method for the humanization of chicken-derived antibodies that relies on CDR transfer to a human VH and VL scaffold with concomitant randomization of Vernier residues defining the CDR orientation (30). Within two consecutive rounds of FACS screening for humanized variants with retained Fc binding, it was possible to select 2 humanized scFv variants. The most abundant single clone (scFv hFc4) was selected for further experiments (**Figure S4**). The protein sequence of the humanized scFv hFc4 (Fv, including CDRs) (**Figure S4**) exhibits a human germline identity of 83.8%, which is comparable to humanized FDA-approved antibodies (79.7-85.5%) (30). The humanized scFv hFc4 was produced in *E. coli* and successfully purified *via* IMAC (**Figure S3B**). Affinity measurement using cetuximab revealed an equilibrium dissociation constant of 20.5 nM (**Figure S3C**), approximately one order of magnitude less affine than its chicken-derived progenitor.

### Design, Generation, and Characterization of Fc-Tamed Trastuzumab Variants

**Figure 3:**
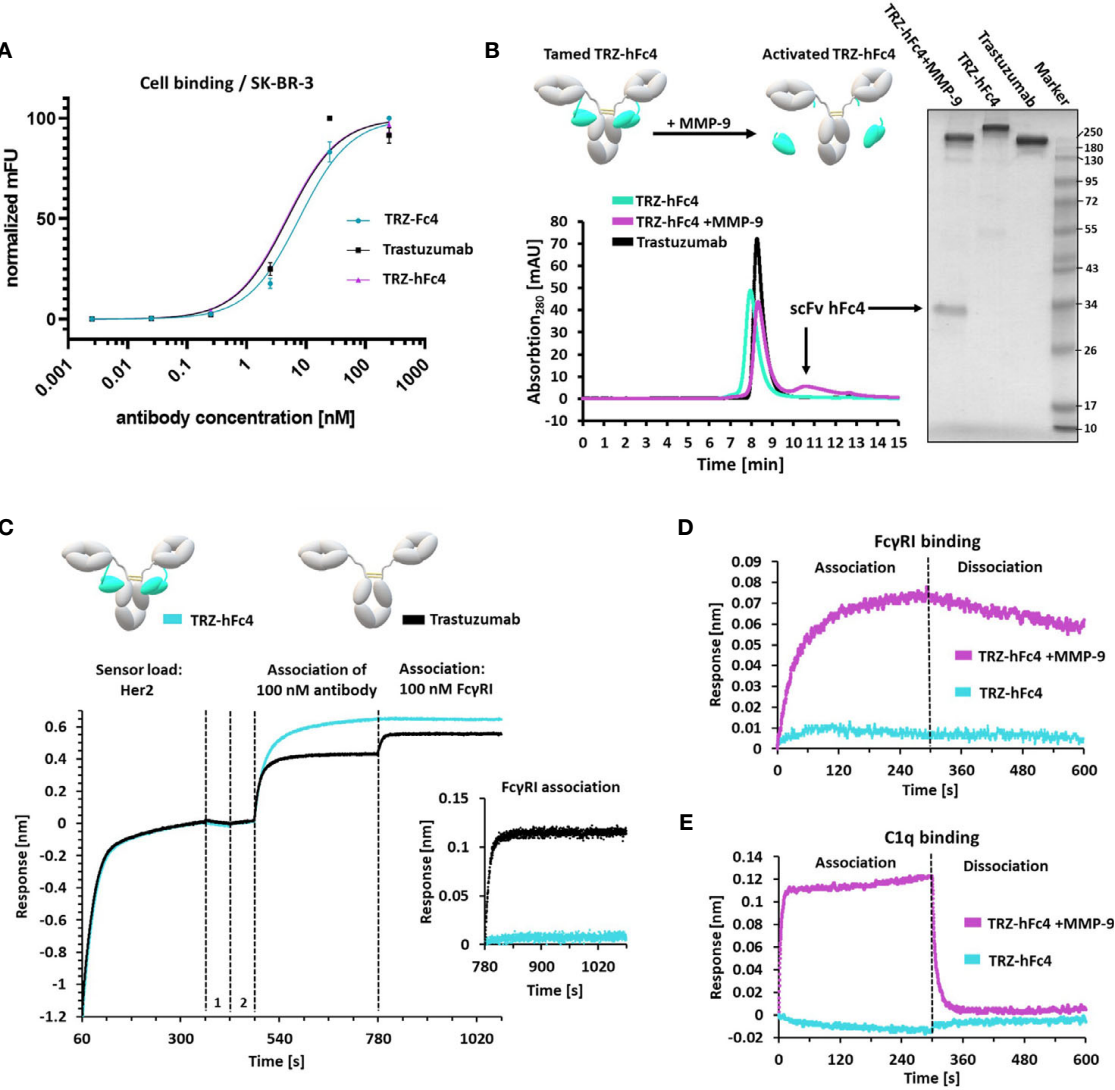
Characterization of tamed Trastuzumab variants TRZ-Fc4, TRZ-hFc4. **(A)** Flow-cytometry analysis using Her2-positive SK-BR-3 cells. Antibody binding was detected using anti-human kappa PE detection antibody. K_D_ (SK-BR-3 cell binding) including 95%-confidence interval analyzed *via* flow-cytometry. Error bars represent standard deviation derived by experimental duplicates. **(B)** Protease cleavage of TRZ-hFc4 by MMP-9 confirmed by SEC and non-reducing SDS-PAGE (4-15% polyacrylamide). **(C)** BLI epitope binning. Biotinylated Her2 was loaded onto SAX sensor tips. Two quenching steps (1, 2) were performed, followed by association of 100 nM trastuzumab or TRZ-hFc4. A subsequent association step was performed using 100 nM FcγRI. A detailed view of the FcγRI association step (second 780-1080) is shown. All association steps were performed in kinetics buffer. **(D)** Association and dissociation of 50 nM FcγRI to TRZ-hFc4 and MMP-9 treated TRZ-hFc4. **(E)** Association and dissociation of 100 nM c1q to TRZ-hFc4 and MMP-9 treated TRZ-hFc4.

Next, we investigated, whether the masking unit can be fused to the therapeutic antibody trastuzumab with a flexible linker retaining Fc masking ability. Moreover, we designed MMP-9 protease recognition motifs into the linker region for tumor-specific cleavage of the fusion and release of the masking unit. To this end, Fc-tamed antibody fusions were generated both with the chicken-derived Fc4 and the humanized version hFc4 (**Figure 1A**). These scFvs were fused to the C-terminus of the antibody light chain *via* an MMP-9 cleavable linker. Using a rational design approach, we identified the longest distance between the C-terminus of the kappa light chain and known Fc amino acid residues, responsible for FcγR interaction (6.44 nm) (**Figure S5**) (3). The same distance calculation was performed for the IgG1 hinge region (Fab to Fc distance) and the commonly used (Gly_4_Ser)_3_ (intra-)scFv linker (RCSB PDB: 1hzh, 5yd3). Then, we calculated the ratios of the spatial distance (nm) and number of amino acid (aa) residues of the hinge region and the (intra-)scFv linker (0.20-0.23 nm/aa). Consequently, we designed a 33 amino acid flexible (G_4_S)-based linker, comprising a ratio of 0.20 nm/aa (**Figure S5**). A tandem protease recognition site (PLGLA), known to be addressed by MMP-9 was included in the linker sequence (35). Three different trastuzumab-based antibody variants were designed, only differing by the respective scFv (Fc4, hFc4, scFv ctrl [non-Fc binding control scFv)]. Consequently, all three antibody constructs (TRZ-Fc4, TRZ-hFc4, TRZ-ctrl) were produced in Expi293F cells and purified *via* affinity chromatography. Purity and protein size was confirmed using non-reducing, as well as reducing SDS-PAGE analysis (**Figures 3B** and **S6**). To further characterize the antibody variants, we performed size exclusion chromatography (SEC) (**Figure S7**). Both antibody variants comprising an Fc-masking domain (TRZ-hFc4, TRZ-Fc4) displayed excellent aggregation behavior (2.2% and 2.6% protein aggregates, respectively) (**Figure S7**), which was comparable to the antibody variant fused with the non-Fc binding control scFv (TRZ-ctrl) (2.7%). We analyzed the thermal stability of the TRZ-hFc4, TRZ-Fc4 and TRZ-ctrl using nano differential scanning fluorimetry. All three proteins exhibited similar melting temperatures (79.7-80.5°C) (**Figure S8**), which is in the range of the melting temperature reported for trastuzumab (36). We used flow cytometry to evaluate the binding characteristics to Her-2 positive SK-BR-3 cells. Both Fc-tamed antibodies and trastuzumab demonstrated comparable binding to SK-BR-3 cells with similar K_D_ values (4.7 – 7.6 nM) indicating that the Fc-masking units do not influence the binding characteristics of the fused antibody (**Figure 3A** and **Table S5**). In order to activate the Fc-tamed antibody variants, TRZ-hFc4 and TRZ-Fc4 proteins were incubated with human MMP-9. Linker proteolysis was confirmed using SDS-PAGE analysis (**Figures 3B** and **S6**), as well as analytic SEC (**Figures 3B** and **S7**). Upon cleavage, both antibody constructs (TRZ-hFc4, TRZ-Fc4) migrated at the same position in the SDS-PAGE gel and displayed the same retention time as trastuzumab during the SEC (**Figures 3B** and **S6**, **S7**), indicating complete linker cleavage. The cleaved scFvs (scFv hFc4, scFv Fc4) migrated corresponding to their respective molecular weight (SI Appendix, **Table S3**). Bio-layer interferometry epitope binning was performed to evaluate, whether the tamed antibody variants (TRZ-hFc4, TRZ-Fc4) bind to their antigen (Her2) and simultaneously impair the binding to FcγRs. Therefore, biotinylated Her2 was immobilized on BLI biosensors, followed by the association of the respective antibody variant and subsequent association of soluble high-affinity FcγRI (100 nM). All antibody constructs demonstrated binding to immobilized Her2. The tamed antibody variants TRZ-hFc4 and TRZ-Fc4 showed no binding signal in presence of 100 nM FcγRI (**Figures 3C** and **S9A**), while both, trastuzumab and TRZ-ctrl showed comparable association to FcγRI (**Figures 3C** and **S9C**). To further evaluate the masking efficacy, we performed BLI using different concentrations of soluble FcγRI (0 - 50 nM) (**Figure S10**). In case of TRZ-hFc4 and TRZ-Fc4, we observed a substantially impaired association of varying concentrations of FcγRI. Upon linker proteolysis of TRZ-hFc4 and TRZ-Fc4 the concentration-dependent FcγRI binding was restored (**Figures 3D** and **S10**) to a level comparable to trastuzumab and TRZ-ctrl. Evaluation of the equilibrium dissociation constants (K_D_) revealed similar values for trastuzumab (1.3 nM), TRZ-ctrl (1.4 nM), the MMP-9 cleaved TRZ-hFc4 (1.2 nM) and MMP-9 cleaved TRZ-Fc4 (1.6 nM) (**Figure S10**). Taking into account that FcγRs and c1q bind to overlapping interaction sites on the Fc domain, affinity measurements with human c1q protein were performed. While TRZ-hFc4 and TRZ-Fc4 showed no detectable binding to varying concentrations of human c1q (0 - 100 nM), trastuzumab and TRZ-ctrl displayed a concentration dependent binding signal (**Figure S11**). Surprisingly, TRZ-ctrl showed an impaired c1q-binding when compared with trastuzumab. At the same time, MMP-9 cleaved TRZ-hFc4 and TRZ-Fc4 demonstrated restored c1q-binding, translating into low nanomolar K_D_ values (**Figures 3E** and **S11**). Furthermore, we validated, whether the masking domains of TRZ-hFc4 and TRZ-Fc4 could interact with other Fc domains in close proximity and at high local concentrations. Therefore, we immobilized Her2 on BLI biosensors, followed by a first association step using 100 nM TRZ-hFc4 or TRZ-Fc4 and a second association step using 500 nM cetuximab (**Figure S12**). No interaction with competing Fc domains was observable. Based on these results, we conduced cell-based assays for further characterization.

### Fc-Tamed Antibody Construct Reduces ADCC Activity and Demonstrates Recovered Antibody Mediated Effector Functions After Activation by MMP-9 Cleavage

**Figure 4:**
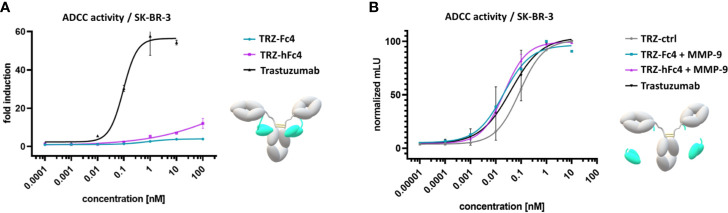
ADCC reporter cell assay using Her2-positive SK-BR-3 cells. **(A)** Cells were incubated with a serial dilution of the Fc-tamed antibody variants TRZ-hFc4 or TRZ-Fc4. Trastuzumab served as a positive control. The assay was performed in the presence of MMP-2/MMP-9 inhibitor. **(B)** Cells were incubated with a serial dilution of MMP-9 cleaved TRZ-hFc4 and TRZ-Fc4 as well as trastuzumab and TRZ-ctrl. Fold induction: ratio of mean luminescence units (mLU) of the respective antibody sample and the blank sample (no antibody). Error bars represent the standard deviation derived from experimental duplicates.

**Figure 5:**
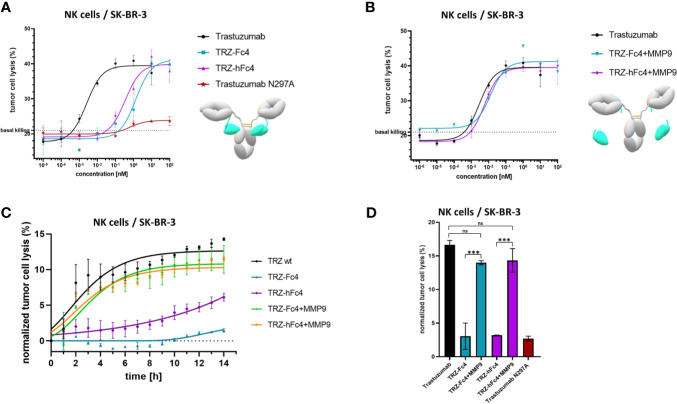
NK cell-mediated tumor cell killing. **(A)** Cells were incubated with a serial dilution of the Fc-tamed antibody variants TRZ-hFc4 or TRZ-Fc4. Trastuzumab served as a positive control. Trastuzumab N297A served as a negative control. Cells were incubated for 12 hours. **(B)** Cells were incubated with a serial dilution of MMP-9 cleaved TRZ-hFc4 and TRZ-Fc4 as well as trastuzumab. Cells were incubated for 12 hours. **(C)** Cells were incubated with 0.01 nM of the respective antibody construct. Tumor cell lysis was determined every 60 minutes. Tumor cell lysis was normalized to the basal killing activity. **(D)** Cells were incubated with 0.01 nM of the respective antibody construct. After 12 hours tumor cell lysis was calculated. One-way ANOVA and Dunnett’s post-hoc multiple comparison testing were performed for statistical analysis. ns (p>0.05); ***(p<0.001). Basal killing, depicted by dashed lines was determined using NK cells mixed with target cells, without the addition of antibody construct. Error bars represent the standard deviation derived from experimental duplicates.

To evaluate, whether our tamed antibody variants are able to diminish antibody-FcγR-mediated cell-cell interaction, a reporter-cell based ADCC assays was performed using Her2-positive SK-BR-3 cells. The ADCC assay was performed in the presence of MMP-2/MMP-9 inhibitor ((2R)-2-[(4-Biphenylylsulfonyl) amino]-3-phenylpropionic acid) since SK-BR-3 cells express and secrete active human MMP-9 and MMP-2 (37, 38). TRZ-hFc4 and TRZ-Fc4 showed significantly lower ADCC induction compared to trastuzumab (**Figure 4A**). At the highest tested concentration (100 nM) TRZ-Fc4 and TRZ-hFc4 mediated only 3.9-fold and 12.1-fold induction respectively, while trastuzumab reached the highest induction level (57.6-fold) already at a 100-fold lower concentration of 1 nM (**Figure 4A**). Comparing the induction level reached by the highest concentration of TRZ-Fc4 (100 nM) with the concentration of trastuzumab required for a similar induction (0.014 nM), we found a 7,100-fold reduction in ADCC activation. Interestingly, the TRZ-hFc4 variant demonstrated only a 2,700-fold reduction (required trastuzumab concentration for similar induction: 0.037 nM), which corresponds to a 2.6-fold lower ADCC reduction compared with TRZ-Fc4. We used MMP-9 cleaved TRZ-hFc4 and TRZ-Fc4 to evaluate if the activation of our Fc-tamed antibodies results in recovered ADCC induction by restored antibody-mediated cell-cell interaction. The cleaved TRZ-Fc4 and TRZ-hFc4 demonstrated recovered ability to induce ADCC (**Figure 4B**), displaying EC_50_ values (EC_50_(TRZ-Fc4) = 2.0 × 10^-2^ nM, EC_50_(TRZ-hFc4) = 2.2 × 10^-2^ nM) comparable to trastuzumab (EC_50_(Trastuzumab) = 3.9 × 10^-2^ nM) and non-cleaved TRZ-ctrl (EC_50_(TRZ-ctrl) = 8.7 × 10^-2^ nM) (**Table S6**). To further validate the functionality of Fc-tamed Trastuzumab variants, cell killing assays using NK cells derived from healthy donors were performed (**Figure 5**). Using SK-BR-3 cells the tamed trastuzumab variants, Trastuzumab induced maximal antibody-dependent NK cell-mediated killing at a concentration of 0.01 nM (EC_50_(Trastuzumab) = 2.5 × 10^-3^ nM). TRZ-Fc4 and TRZ-hFc4 exhibited EC_50_ values of 1.3 nM and 0.34 nM, respectively (**Figure 5** and **Table S7**). The MMP-9 cleaved TRZ-hFc4 and TRZ-Fc4 exhibited EC_50_ values and maximal tumor cell lysis similar to Trastuzumab (**Figures 5B**, **D** and **Table S7**). To evaluate whether the Fc-tamed Trastuzumab variants exhibit a distinct time-dependent activation, antibody-mediated cell killing was determined every 60 minutes over a period of 14 hours (**Figure 5C**). To further evaluate the functionality of Fc-tamed antibodies, MDA-MB-453 cells, with a moderate Her2-expression profile were incubated with 100 nM of the respective antibody (**Figure S13**). Both TRZ-hFc4 and TRZ-Fc4 demonstrated significantly reduced ADCC activation, while the MMP-9 cleaved antibodies showed ADCC activation comparable to Trastuzumab (**Figure S13**).

## Discussion

In order to prevent undesired FcγR binding or complement fixation of therapeutic antibodies, several successful strategies were conducted over the past decades, eventually resulting in fully or partially Fc-silenced antibody therapeutics approved for clinical use (39). However, all reported approaches contribute to a permanently altered Fc domain, leading to permanently impaired antibody-dependent effector functions. In this study, we present a novel antibody format, introducing a masking unit to prevent antibody binding by FcγR-bearing cells or complement components. The Fc-tamed antibodies can be activated by a tumor-specific protease (MMP-9), displaying recovered ability to interact with complement components and FcγR-bearing cells. We used yeast surface display in combination with chicken immunization and multiparameter FACS to generate two anti-isotypic masking domains (anti-IgG1 scFvs), originating from the same antibody clone, displaying different affinities and targeting a FcγR-overlapping epitope. When fused to the light chain of trastuzumab, both masking units reduced the antibody-FcγR-mediated cell-cell interaction by a factor of 2,700 (scFv hFc4) to 7,100 (scFv Fc4) during 6 h incubation. These findings are comparable with recently published data regarding paratope-masking units (23). When using donor-derived NK cells, both Fc-tamed antibodies showed significantly impaired effector functioning (**Figure 5**). At elevated antibody concentrations, both Fc-tamed antibodies induced maximal killing, comparable with Trastuzumab (**Figure 5A**). After MMP-9 mediated activation, both antibody variants induced tumor cell killing comparable to trastuzumab.

The more affine scFv Fc4-based masking unit led to a 2.6-fold stronger reduction of ADCC reporter cell activation compared with the scFv hFc4-based masking unit. The masking efficiency can be directly traced back to the masking domain affinity, since both masking domains originate from the same antibody clone, comprising the same CDRs, targeting the same epitope and exhibiting similar molecule size. Both Fc-tamed trastuzumab variants were produced successfully, demonstrating unaffected protein characteristics, including aggregation behavior and thermal stability (**Figures S7**, **S8**). Upon protease-mediated activation, they were able to induce ADCC, comparable to trastuzumab, indicating that the masking units do not negatively influence protein folding and glycosylation during expression (**Figures 4B, C**). We used a rational design approach to determine a suitable linker, connecting the anti-isotypic scFv with the C-terminus of the light chain. Both tamed antibodies show significant reduction in FcγRI-binding (**Figure S10**) and do not tend to multimeric aggregation (**Figure S7**). This implicates an appropriate length and flexibility of the linker sequence, enabling both masking units to bind the Fc efficiently. However, based on published data (40), we argue that a library-based approach for linker design could result in an improved linker structure, potentially enhancing the masking efficiency even further. We performed Bio-layer interferometry experiments to analyze the masking efficiency which revealed that the association of the high-affinity FcγRI was substantially impaired by both masking domains. However, we still observed a weak residual binding signal (**Figures 3C** and **S10**). Evaluating the masking efficiency of TRZ-hFc4 and TRZ-Fc4 regarding c1q association no binding was observed (**Figures 3D** and **S11**). This could be explained by possible steric hindrance, due to the expanded molecular size of the hexameric c1q protein (410 to 462 kDa), compared to FcγRI (50-65 kDa). However, MMP-9 cleaved TRZ-hFc4 and TRZ-Fc4 demonstrated recovered c1q binding. TRZ-ctrl, displayed unaffected FcγRI-binding. C1q binding was substantially impaired (**Figure S11**). Published data suggests that even small changes in the Fab-Fc orientation can lead to impaired accessibility by c1q (2). Additionally, TRZ-ctrl demonstrated a distinct retention time during analytical SEC (**Figure S7**), indicating an enlarged hydrodynamic radius, possibly originating from the non-binding, exposed scFv fused to the light chain. Recent strategies regarding paratope masking of antibodies either contribute to steric hindrance by non-specific masking units or anti-idiotypic masking units. Our approach contributes to anti-isotypic masking units (anti-IgG1). The masking scFv demonstrated potent FcγR-blocking properties using cetuximab (**Figure 2C**), rituximab (**Figure 2D**) as well as trastuzumab (**Figure 4A**). Therefore, this newly developed antibody format can be applied to different antibodies, by simply changing the Fab domains. We used *in vitro* reporter cell-based assays to investigate the reduced interaction to FcγRs. However, to further characterize the influence of the Fc-tamed antibodies on rare Fc-mediated effects, including potentially reduced thrombocytopenia, trogocytosis, immune complex clearance and activation of CD32b-positive scavenger endothelial cells, *in vivo* studies should be considered (41). Therapeutic antibodies targeting immune checkpoints, typically comprise an IgG4 Fc or an engineered IgG1 Fc with reduced FcγR and c1q binding, which prevents undesired ADCC, CDC, immune complex clearance and cytokine release mediated by FcγR cross-linking (42). However, anti-PD-L1 antibodies, which comprise a functional IgG1-Fc, demonstrate improved efficacy *in vivo* (43), which was linked to antibody-mediated effector functions against PD-L1 positive tumor cells (44). We argue that the isotype switch of IgG4 to Fc-tamed IgG1 immune checkpoint targeting antibodies could potentially lead to enhanced potency, combining checkpoint blockade mechanisms and tumor proximity induced antibody-mediated effector functions. We used MMP-9 as a model protease. However, when targeting different cancer types, screening for suitable tumor-specific proteases and the use of respective cleavable polypeptide linkers potentially improve the tumor-proximity induced activation of the Fc-tamed antibodies.

It is known that antibody glycoengineering, particularly removal of the core fucosylation, significantly enhances ADCC and ADCP (45, 46). Combining glycoengineering with the newly developed Fc-tamed antibody format, potentially enhances Fc-mediated effector functioning while reducing undesired FcγR and complement related adverse effects. Additionally, mutations within the Fc part can significantly enhance particular effector functions. A combination of Fc-masking and effector-enhanced, glycoengineered antibodies could result in a synergistic effect, simultaneously reducing potential systemic toxicity (47). In conclusion, our approach represents a generalizable platform, based on an IgG1-Fc-tamed antibody, demonstrating impaired interaction with FcγR and c1q and regaining full effector function mediated efficacy upon tumor-associated protease cleavage. Since therapeutic application of antibodies is limited, due to unwanted interactions to healthy cells and Fc-receptor bearing cells, this type of antibody format potentially contributes to a safer and more controllable drug profile.

## Data Availability Statement

The original contributions presented in the study are included in the article/**Supplementary Material**, further inquiries can be directed to the corresponding author/s.

## Ethics Statement

Ethical review and approval was not required for the animal study because Animal immunization (Chicken/Gallus gallus domesticus) was performed by Davids Biotechnologie GmbH (Regensburg). Experimental procedures and animal care were in accordance with EU animal welfare protection laws and regulations.

## Author Contributions

AE and HK conceived and designed the experiments. AE, DY, DF, and KH performed the experiments. AE, DY, and HK analyzed the data. SB and UB gave scientific advice. AE and HK wrote the manuscript. All authors contributed to the article and approved the submitted version.

## Funding

This work was supported by the MerckLab@TU Darmstadt, by the Department of Protein Engineering and Antibody Technologies at Merck KGaA, Darmstadt and by Deutsche Forschungsgemeinschaft through grant KO 1390/14-1. We acknowledge support by the Deutsche Forschungsgemeinschaft (DFG – German Research Foundation) and the Open Access Publishing Fund of Technical University of Darmstadt. The funder was not involved in the study design, collection, analysis, interpretation of data, the writing of this article or the decision to submit it for publication.

## Conflict of Interest

AE is employed by TU Darmstadt in frame of a collaboration project with Merck KGaA. DY is employed by TU Darmstadt in frame of a collaboration project with Merck Healthcare KGaA. KH, SB, and UB are employed by Merck Healthcare KGaA. DF was employed by TU Darmstadt in frame of a collaboration project with Ferring Darmstadt Laboratory. HK is employed by TU Darmstadt.

## Publisher’s Note

All claims expressed in this article are solely those of the authors and do not necessarily represent those of their affiliated organizations, or those of the publisher, the editors and the reviewers. Any product that may be evaluated in this article, or claim that may be made by its manufacturer, is not guaranteed or endorsed by the publisher.
