# Supplementary material for: Protease-Activation of Fc-Masked Therapeutic Antibodies to Alleviate Off-Tumor Cytotoxicity
Source: Front Immunol. 2021 Aug 3;12:715719. doi: 10.3389/fimmu.2021.715719 (PMC8369199; doi:10.3389/fimmu.2021.715719)
Supplement: Supplementary file 1 [file DataSheet_1.pdf]

Supplementary Material

1 Supplementary Data

Table S1. Staining strategy for all yeast surface display sorting rounds and single clone analysis including dilution of primary and secondary labeling agents.

	**scFv-surface presentation**		**Fc-Binding**		**FcγRI-Binding**	
**Sorting round**	**Primary labeling agent**	**Secondary labeling agent**	**Primary labeling agent**	**Secondary labeling agent**	**Primary labeling agent**	**Secondary labeling agent**
**1st Round (Fc-binding scFvs)**	anti-c-myc biotin antibody (1:50)	Streptavidin-PE (1:200)	1 µM Fc-protein-dylight650	-	-	-
**2nd Round (Fc-binding scFvs)**	anti-c-myc biotin antibody (1:50)	Streptavidin-PE (1:200)	1 µM Fc-protein-dylight650	-	-	-
**3rd Round (Fc-binding scFvs)**	anti-c-myc biotin antibody (1:50)	Streptavidin-PE (1:200)	500 nM Fc-protein-dylight650	-	-	-
**1st Round (FcγR blocking scFvs)**	-	-	1 µM Cetuximab-dylight650	Goat anti-Human IgG Fc Secondary Antibody PE (1:80)	500 nM FcγRI-His-tag	6x-His Tag monoclonal antibody Alexa Fluor 647
**2nd Round (FcγR blocking scFvs)**	-	-	1 µM Cetuximab-dylight650	Goat anti-Human IgG Fc Secondary Antibody PE (1:80)	500 nM FcγRI-His-tag	6x-His Tag monoclonal antibody Alexa Fluor 647
**3rd Round (FcγR blocking scFvs)**	-	-	1 µM Cetuximab-dylight650	Goat anti-Human IgG Fc Secondary Antibody PE (1:80)	500 nM FcγRI-His-tag	6x-His Tag monoclonal antibody Alexa Fluor 647

**Single clone analysis**	-	-	500 nM Cetuximab-dylight650	Goat anti-Human IgG Fc Secondary Antibody PE (1:80)	500 nM FcγRI-His-tag	6x-His Tag monoclonal antibody Alexa Fluor 647
**1st Round (Humanization)**	anti-c-myc biotin antibody (1:50)	Streptavidin-APC (1:75)	500 nM Cetuximab	Goat anti-Human IgG Fc Secondary Antibody PE (1:80)	-	-
**2nd Round (Humanization)**	anti-c-myc biotin antibody (1:50)	Streptavidin-APC (1:75)	500 nM Cetuximab	Goat anti-Human IgG Fc Secondary Antibody PE (1:80)	-	-
**Single clone analysis (Humanization)**	Anti-c-myc FITC (1:25)	-	500 nM Cetuximab-dylight650	-	-	-

Table S2. Staining procedure prior to yeast cell sorting. 1×10^7 yeast cells were stained in a total volume of 20 µl. All incubation steps and wash steps were performed at 4 °C.

**Sorting Round**	**Incubation with target proteins in PBS-B pH 7.4, 30 minutes, 4 °C**	**Wash step with 1ml PBS-B pH 7.4, 4 °C**	**Primary labeling agent for surface display detection in PBS-B pH 7.4, 15 min, 4 °C**	**Wash step with 1ml PBS-B pH 7.4, 4 °C**	**Secondary labeling agent for surface detection and target protein detection in PBS-B pH 7.4, 15 min, 4 °C**	**Wash step with 1ml PBS-B pH 7.4, 4 °C**
**1st Round (Fc-binding scFvs)**	+	1x	+	1x	+	2x
**2nd Round (Fc-binding scFvs)**	+	1x	+	1x	+	2x
**3rd Round (Fc-binding scFvs)**	+	1x	+	1x	+	2x

**1st Round (FcγR blocking scFvs)**	+	1x	-	1x	+	2x
**2nd Round (FcγR blocking scFvs)**	+	1x	-	1x	+	2x
**3rd Round (FcγR blocking scFvs)**	+	1x	-	1x	+	2x
**Single clone analysis**	+	1x	+	1x	+	2x
**1st Round (Humanization)**	+	1x	+	1x	+	2x
**2nd Round (Humanization)**	+	1x	+	1x	+	2x
**Single clone analysis (Humanization)**	+	1x	+	1x	+	2x

Table S3. Calculated molecular protein weights (kDa) under reducing and non-reducing conditions as well as before and after MMP-9 cleavage.

	**TRZ-hFc4**	**TRZ-Fc4**	**TRZ-ctrl**	**Trastuzumab**
**Non-reduced**	202.8 kDa (2HC+2LC-scFv)	202.0 kDa (2HC+2LC-scFv)	203.0 kDa (2HC+2LC-scFv)	145.4 kDa (2HC+2LC)
**Reduced**	49.3 kDa (HC)<br>52.1 kDa (LC-scFv)	49.3 kDa (HC)<br>51.7 kDa (LC-scFv)	49.3 kDa (HC)<br>52.3 kDa (LC-scFv)	49.3 kDa (HC)<br>23.4 kDa (LC)
**Non-reduced and MMP-9 cleaved**	147.1 kDa (2HC+2LC)<br>27.0 kDa (scFv)	147.1 kDa (2HC+2LC)<br>26.7 kDa (scFv)	-	-
**Reduced and MMP-9 cleaved**	49.3 kDa (HC)<br>24.3 kDa (LC)<br>27.0 kDa (scFv)	49.3 kDa (HC)<br>24.3 kDa (LC)<br>26.7 kDa (scFv)	-	-

Table S4. Protein sequence information of scFv Fc4, scFv hFc4, scFv ctrl and light chain sequences TRZ-Fc4, TRZ-hFc4 and TRZ-ctrl.

Molecule	Protein Sequence
**scFv Fc4 (Fc-specific, chicken)**	AVTLDESGGGLQTPGGALSLVCKASGFTFSSYAMNWVRQAPGKGLEFVAGINAAGSGANYAPAVKGRATISRDNGQRTVRLQLSNLRAEDTATYYCAKG<br>SGSCNHCGGGHGGMIDEWGHGTEVIVSSGGGGSGGGGSGGGGSALTQPSSVSANPGETVKITCSGGSSTYYGWYQQKSPGTAPVTVIYWDDERPSNIPSRF<br>SGSASGSTNTLTITGVQVEDEAVYFCGGYDSSVGIFGAGTTLTVL
**scFv-ctrl (chicken)**	ALTLDESGGGLQTPGGGLSLVCKASGFDFSSYAMIWVRQAPGKGLEYVAGINDDGSYTYYAPAVKGRATISRDDGQSTVRLQVNSLKAEDTATYYCTKSA<br>GPWTYPGGIDAWGHGTEVIVSSGGGGSGGGGSGGGGSALTQPSSVSANPGETVKITCSGGYSDDGSYYYYGWFQQKSPGSAPVTVIYDNTNRPSNIPSRFSG<br>STSGSTSTLTITGVRAEDEAVYYCGSIDTGYAGIFGAGTTLTVL
**scFv hFc4 (Fc-specific, humanized)**	EVQLLESGGGLVQPGGSLRLSCAASGFTFSSYAMNWVRQAPGKGLEFVAGINAAGSGANYAPAVKGRATISRDNSPNTVYLQMNSLRAEDTAVYYCAKGS<br>GSCNHCGGGHGGMIDEWGQGTLVTVSSGGGGSGGGGSGGGGSSYELMQPPSVSVSPGQTARITCSGGSSTYYGWYQQKPGQAPVTVIYWDDERPSGIPER<br>FSGSSSGNTVTLTINGVQAEDEADYYCGGYDSSVGIFGGGTKLTVL
**TRZ-Fc4 LC (LC-scFv)**	DIQMTQSPSSLSASVGDRVTITCRASQDVNTAVAWYQQKPGKAPKLLIYSASFLYSGVPSRFSGSRSGTDFTLTISSLQPEDFATYYCQQHYTTPPTFGQGTK<br>VEIKRTVAAPSVFIFPPSDEQLKSGTASVVCLLNNFYPREAKVQWKVDNALQSGNSQESVTEQDSKDSTYSLSSTLTLSKADYEKHKVYACEVTHQGLSSPV<br>TKSFNRGECGKSGGGGSPLGLAGGGGGSPLGLAGGGGGSALRAVTLDESGGGLQTPGGALSLVCKASGFTFSSYAMNWVRQAPGKGLEFVAGINAAGSG<br>ANYAPAVKGRATISRDNGQRTVRLQLSNLRAEDTATYYCAKGSGSCNHCGGGHGGMIDEWGHGTEVIVSSGGGGSGGGGSGGGGSALTQPSSVSANPGET<br>VKITCSGGSSTYYGWYQQKSPGTAPVTVIYWDDERPSNIPSRFSGSASGSTNTLTITGVQVEDEAVYFCGGYDSSVGIFGAGTTLTVLGSWSHPQFEK
**TRZ-hFc4 LC (LC-scFv)**	DIQMTQSPSSLSASVGDRVTITCRASQDVNTAVAWYQQKPGKAPKLLIYSASFLYSGVPSRFSGSRSGTDFTLTISSLQPEDFATYYCQQHYTTPPTFGQGTK<br>VEIKRTVAAPSVFIFPPSDEQLKSGTASVVCLLNNFYPREAKVQWKVDNALQSGNSQESVTEQDSKDSTYSLSSTLTLSKADYEKHKVYACEVTHQGLSSPV<br>TKSFNRGECGKSGGGGSPLGLAGGGGGSPLGLAGGGGGSALREVQLLESGGGLVQPGGSLRLSCAASGFTFSSYAMNWVRQAPGKGLEFVAGINAAGSGA<br>NYAPAVKGRATISRDNSPNTVYLQMNSLRAEDTAVYYCAKGSGSCNHCGGGHGGMIDEWGQGTLVTVSSGGGGSGGGGSGGGGSSYELMQPPSVSVSPG<br>QTARITCSGGSSTYYGWYQQKPGQAPVTVIYWDDERPSGIPERFSGSSSGNTVTLTINGVQAEDEADYYCGGYDSSVGIFGGGTKLTVLGSWSHPQFEK
**TRZ-ctrl LC (LC-scFv)**	DIQMTQSPSSLSASVGDRVTITCRASQDVNTAVAWYQQKPGKAPKLLIYSASFLYSGVPSRFSGSRSGTDFTLTISSLQPEDFATYYCQQHYTTPPTFGQGTK<br>VEIKRTVAAPSVFIFPPSDEQLKSGTASVVCLLNNFYPREAKVQWKVDNALQSGNSQESVTEQDSKDSTYSLSSTLTLSKADYEKHKVYACEVTHQGLSSPV<br>TKSFNRGECGKSGGGGSPLGLAGGGGGSPLGLAGGGGGSALRALTLDESGGGLQTPGGGLSLVCKASGFDFSSYAMIWVRQAPGKGLEYVAGINDDGSYT<br>YYAPAVKGRATISRDDGQSTVRLQVNSLKAEDTATYYCTKSAGPWTYPGGIDAWGHGTEVIVSSGGGGSGGGGSGGGGSALTQPSSVSANPGETVKITCSG<br>GYSDDGSYYYYGWFQQKSPGSAPVTVIYDNTNRPSNIPSRFSGSTSGSTSTLTITGVRAEDEAVYYCGSIDTGYAGIFGAGTTLTVLGSWSHPQFEK

Table S5. K_D values (SK-BR-3 cell binding) including 95%-confidence interval analyzed via flow-cytometry. Flow-cytometry analysis was performed using Her2-positive SK-BR-3 cells. Values were calculated based on experimental duplicates.

	**TRZ-hFc4**	**TRZ-Fc4**	**Trastuzumab**
**K_D [nM]** (SK-BR-3)	**4.7**<br>(3.1-7.0)	**7.6**<br>(6.0 -9.8)	**4.9**<br>(3.1 -7.6)

Table S6. EC_{50} values (ADCC activity) including 95%-confidence interval derived from the ADCC reporter cell assay using Her2-positive SK-BR-3 cells. Values were calculated based on experimental duplicates.

	TRZ-hFc4	TRZ-Fc4	Trastuzumab	TRZ-hFc4 +MMP-9 (activated)	TRZ-Fc4 +MMP-9 (activated)	TRZ-ctrl
**EC_{50} [nM]** **(ADCC/ SK-BR-3)**	**n.c.**	**n.c.**	**3.9×10^{-2}** (1.2×10^{-2} – 2.1×10^{-1})	**2.2×10^{-2}** (1.7×10^{-2} – 2.6×10^{-2})	**2.0×10^{-2}** (1.4×10^{-2} – 3.1×10^{-2})	**8.7×10^{-2}** (8.5×10^{-2} – 8.9×10^{-2})

Table S7. EC_{50} values including 95%-confidence interval derived from the NK cell-based ADCC assay using Her2-positive SK-BR-3 cells. Values were calculated based on experimental duplicates.

	TRZ-hFc4	TRZ-Fc4	Trastuzumab	TRZ-hFc4 +MMP-9 (activated)	TRZ-Fc4 +MMP-9 (activated)	Trastuzumab N297A
**EC_{50} [nM]** **(NK cell mediated killing)**	**0.34** (0.14–0.78)	**1.3** (0.5– 3.1)	**2.5×10^{-3}** (1.3×10^{-3} – 4.9×10^{-3})	**5.8×10^{-3}** (3.9×10^{-3} – 8.3×10^{-3})	**1.2×10^{-2}** (5.5×10^{-3} – 3.1×10^{-2})	**n.c.**

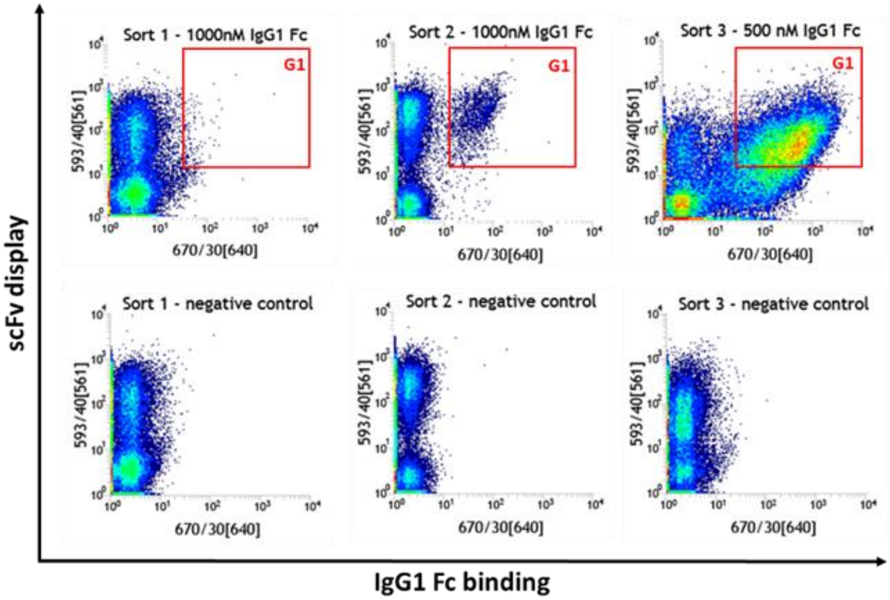

Figure S1. Cell sorting rounds for the isolation of yeast cells displaying a Fc-binding scFv. Surface presentation is plotted on the y-axis and Fc-binding on the x-axis. 50,000 cells are displayed. Negative controls represent samples without Fc protein. Each round, cells located in gate 1 (G1) (scFv displaying and simultaneous Fc-binding cells) were isolated. A detailed staining strategy is summarized in Table S1 and Table S2.

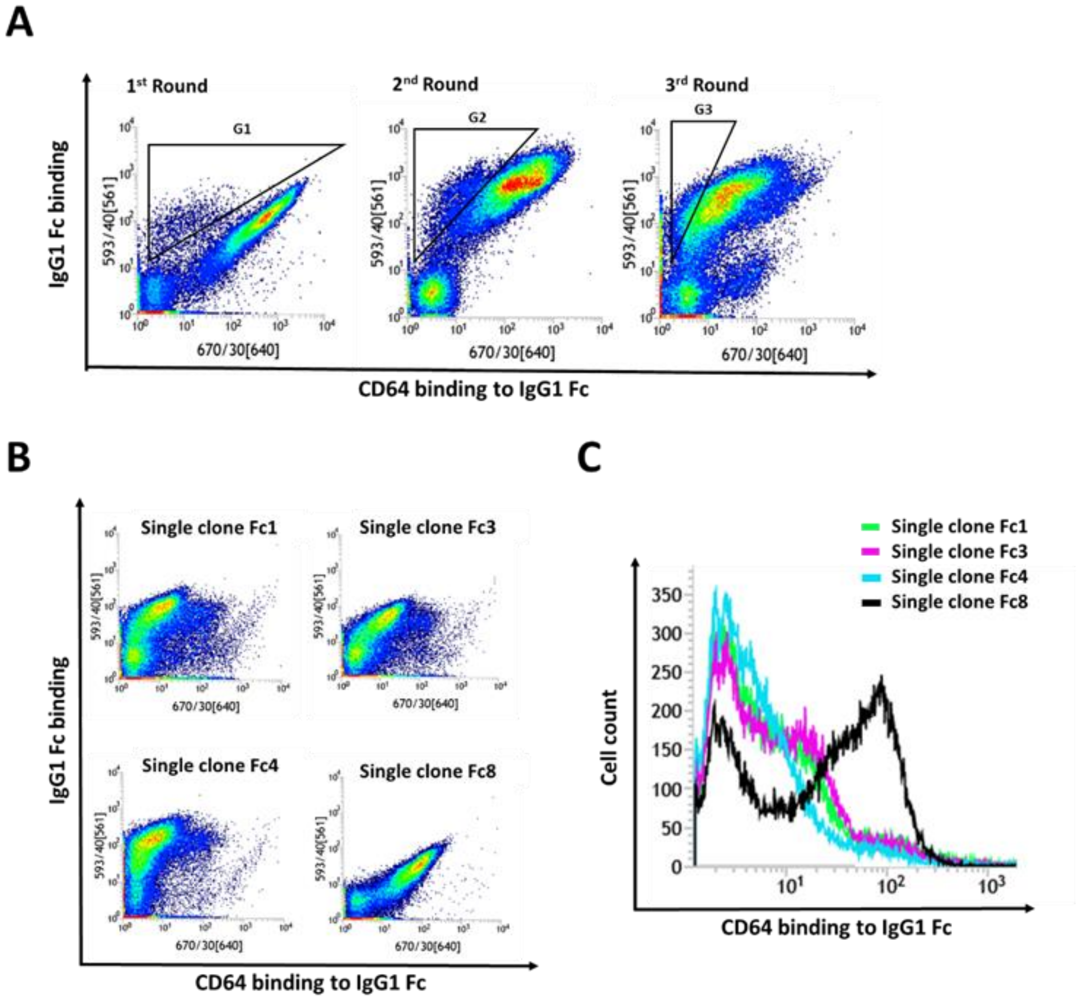

Figure S2. Cell sorting rounds and single clone analysis for the isolation of yeast cells presenting a Fc-binding scFv, while the scFv blocks the binding to soluble FcγRI (CD64). (**A**) Three rounds of FACS for the enrichment of Fc-binding and FcγRI-blocking scFv presenting cells located in gate 1(G1), gate 2 (G2) and gate 3 (G3), respectively. Yeast cells were incubated with 1000 nM cetuximab and 500 nM FcγRI. (**B**) Yeast single clone analysis. Cells were incubated with 500 nM cetuximab and 500 nM FcγRI. Fc-binding is plotted on the y-axis and FcγRI (CD64)-binding on the x-axis. 50,000 cells are displayed. (**C**) Histogram overlay (CD64-binding) of four yeast single clones. A detailed staining strategy is summarized in Table S1 and Table S2.

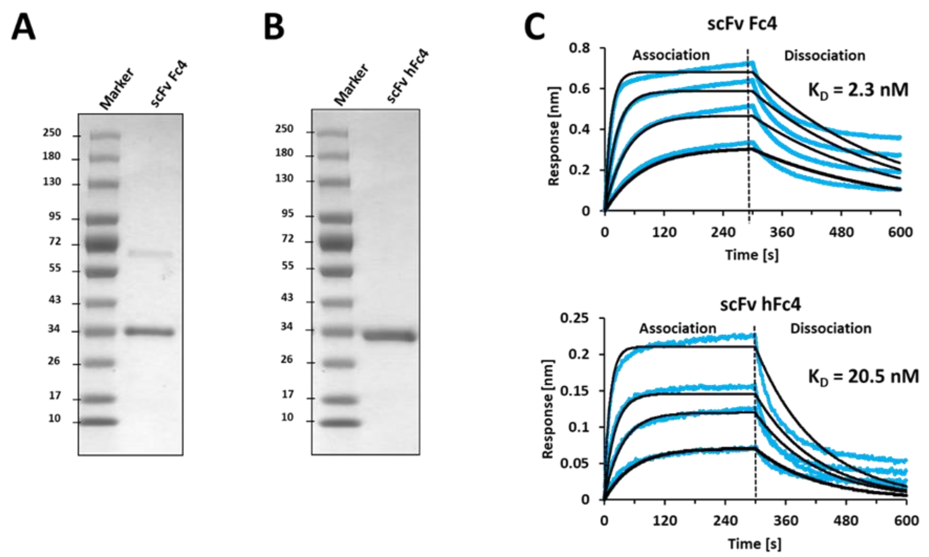

Figure S3. SDS-PAGE and affinity measurements of scFv Fc4 and scFv hFc4. SDS-PAGE of scFv Fc4 (**A**) and scFv hFc4 (**B**). 3 µg of protein were analyzed under reducing conditions. (**C**) Determination of binding kinetics using BLI. Cetuximab was loaded to anti-human Fab-CH1 Biosensors (Fab2G) biosensor tips and probed with varying concentrations of the respective scFv (scFv Fc4: 6.25, 12.5, 25, 50 nM; scFv hfc4: 25, 50, 100, 200 nM). All steps were performed using kinetics buffer. Equilibrium dissociation constants (K_D) were determined based on Savitzky-Golay filtering and a 1:1 Langmuir binding model.

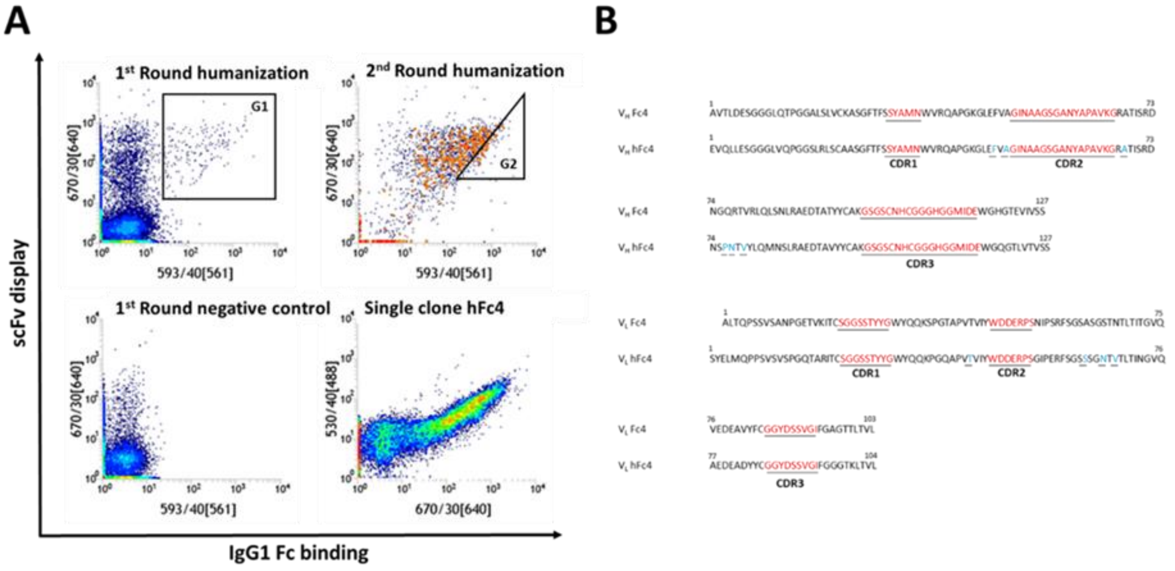

Figure S4. Cell sorting rounds and single clone analysis for the isolation of yeast cells presenting a humanized variant of scFv Fc4. (**A**) Two consecutive sorting rounds for yeast cells presenting a humanized variant of scFv Fc4 binding to 500 nM cetuximab. Cells located in gate 1 (G1) and gate 2 (G2) were isolated. The negative control represents the cell population stained without Fc protein. Surface presentation is plotted on the y-axis and Fc-binding on the x-axis. A detailed staining strategy is summarized in Table S1 and Table S2. (**B**) Protein sequence alignment of the VH and VL domain of scFv Fc4 and scFv hFc4. Complementary-determining regions (CDR1, CDR2, CDR3) are highlighted in red. Vernier residues are highlighted in blue.

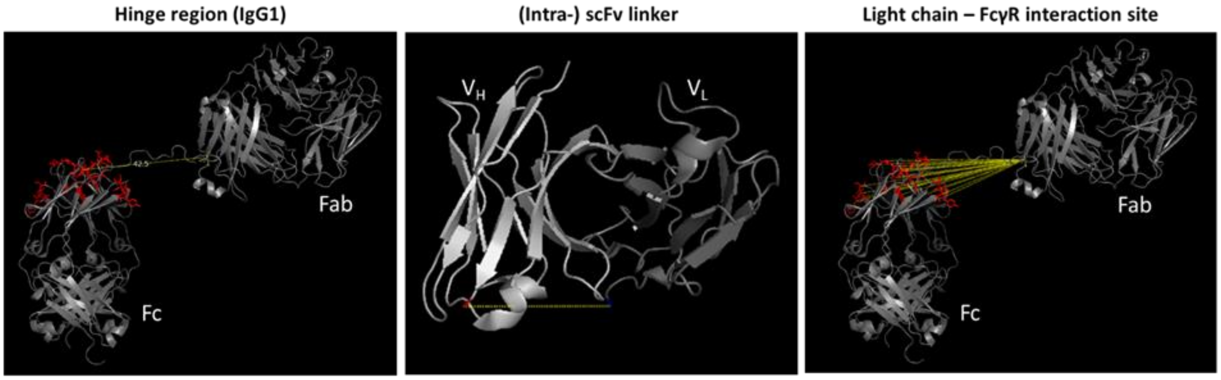

	Number of amino acids	Protein sequence	Maximum distance [nm]	Ratio [nm/aa]
**Hinge region (IgG1)**	21	EPKSCDKTHTCPPCPAPELLG	4.25	0.20
**(Intra-) scFv linker**	15	GGGGSGGGGSGGGGS	3.39	0.23
**Light chain-linker**	33	GKSGGGGSPLGLAGGGGGSPLGLAG GGGGSALR	6.44	0.20

Figure S5. Conceptual linker design for the construction a Fc-tamed antibody. Inter-residue distances were determined using Pymol. The distance between the Fab domain and the Fc domain (left panel) of the (intra-) scFv linker (middle panel) and the longest distance between the C-terminus of the light chain and residues known to be essential for FcγR binding were calculated. The number of amino acids, the respective protein sequence and the corresponding maximum distances are listed as well as the ratio of amino acids divided by the maximum distance (nm/aa). Based on these data the 33 amino acid long light chain-linker was designed.

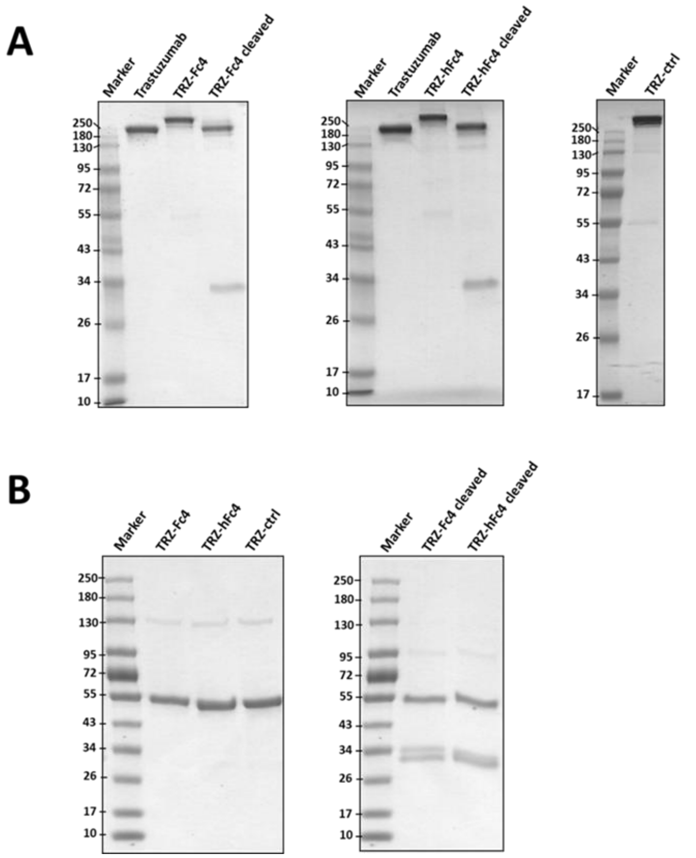

Figure S6. SDS-PAGE (4-15% Polyacrylamide) of TRZ-Fc4, TRZ-hFc4 and TRZ-ctrl. In addition, TRZ-Fc4 and TRZ-hFc4 were treated with MMP-9. (**A**) Non-reducing SDS-PAGE. Trastuzumab was analyzed enabling comparison between non-cleaved and MMP-9-cleaved TRZ-Fc4 and TRZ-hfc4. (**B**) Reducing SDS-PAGE. 3 µg of each protein were analyzed. Molecular weights are listed in Table S3.

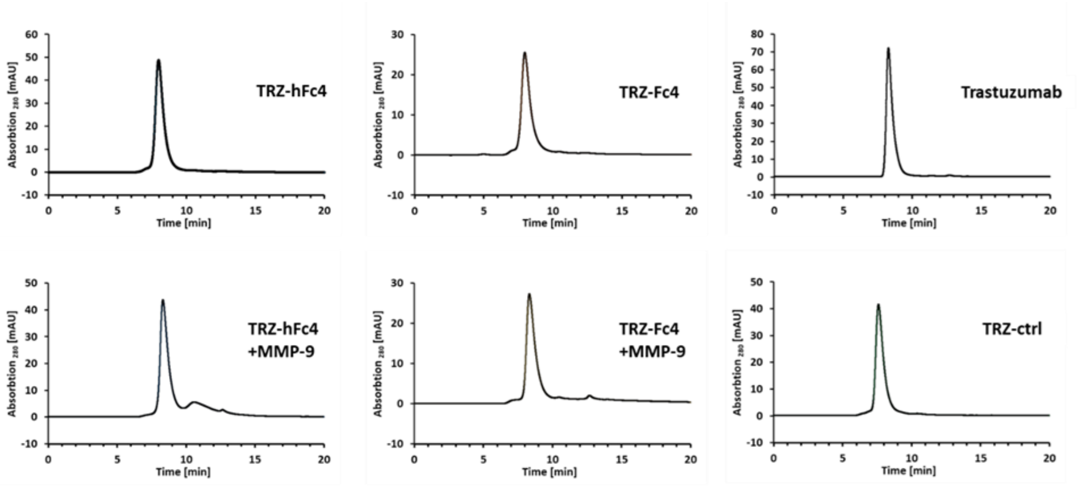

	TRZ-hFc4	TRZ-Fc4	TRZ-hFc4 +MMP-9	TRZ-Fc4 +MMP-9	Trastuzumab	TRZ-ctrl
**Retention Time [min]**	8.0	8.0	8.3	8.3	8.3	7.6
**Aggregates [%]**	2.2	2.6	n.c.	n.c.	n.d.	2.7

Figure S7. Size exclusion chromatography of TRZ-Fc4, TRZ-hFc4 and TRZ-ctrl. In addition, TRZ-Fc4 and TRZ-hFc4 were treated with MMP-9. Trastuzumab was analyzed enabling comparison between non-cleaved and MMP-9-cleaved TRZ-Fc4 and TRZ-hFc4. n.c. = not calculated; n.d.= not detectable. Retention times and aggregates (AUC) were calculated using Agilent Technologies 1260 Infinity system software.

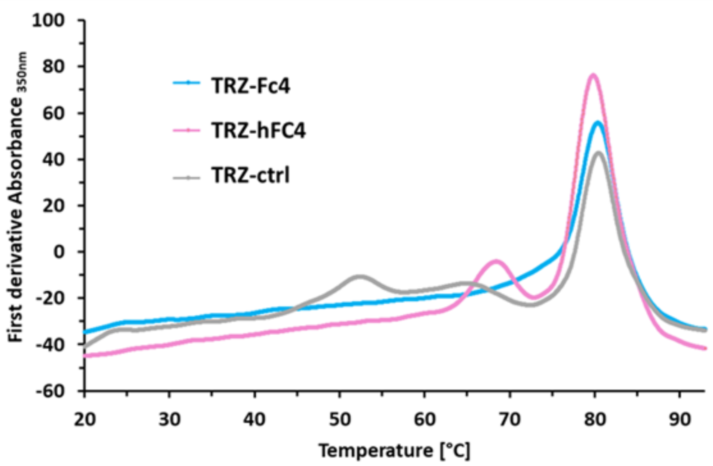

	TRZ-hFc4	TRZ-Fc4	TRZ-ctrl
Melting point [°C]	79.9	80.3	80.4

Figure S8. Determination of thermal stability of TRZ-Fc4, TRZ-hFc4 and TRZ-ctrl using nano differential scanning fluorimetry measurement. First derivative of the fluorescence at 350 nm was calculated and plotted in relation to a 1 °C/min temperature gradient. Data analysis and melting point calculation was performed using Prometheus ThermControl version v.2.1.2.

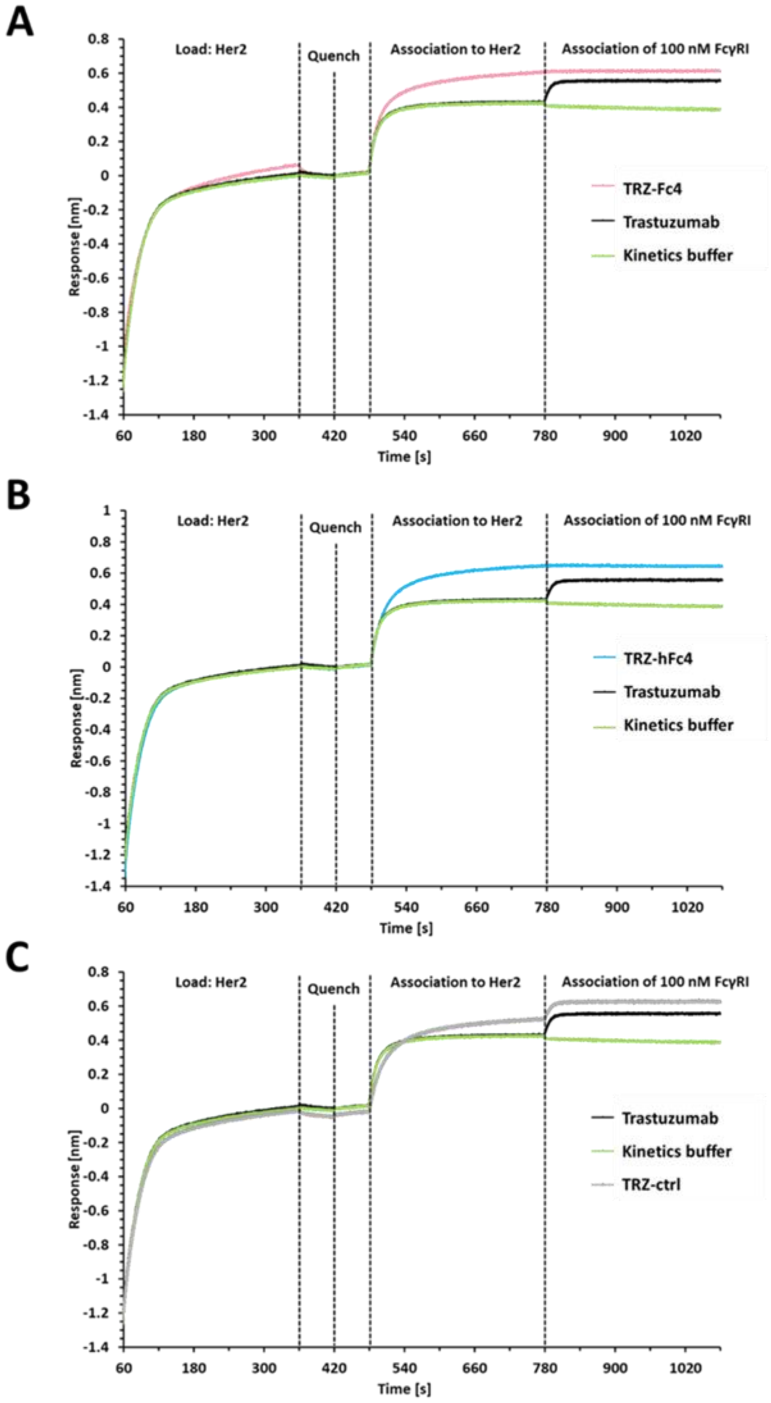

Figure S9. Epitope binning of TRZ-Fc4, TRZ-hFc4, TRZ-ctrl and trastuzumab. SAX biosensors were loaded with biotinylated Her2 (Load: Her2), followed by two quenching steps. Subsequently, 100 nM TRZ-Fc4 (**A**), TRZ-hFc4 (**B**) or TRZ-ctrl (**C**) were applied (Association to Her2), followed by a second association step using 100 nM FcγRI.

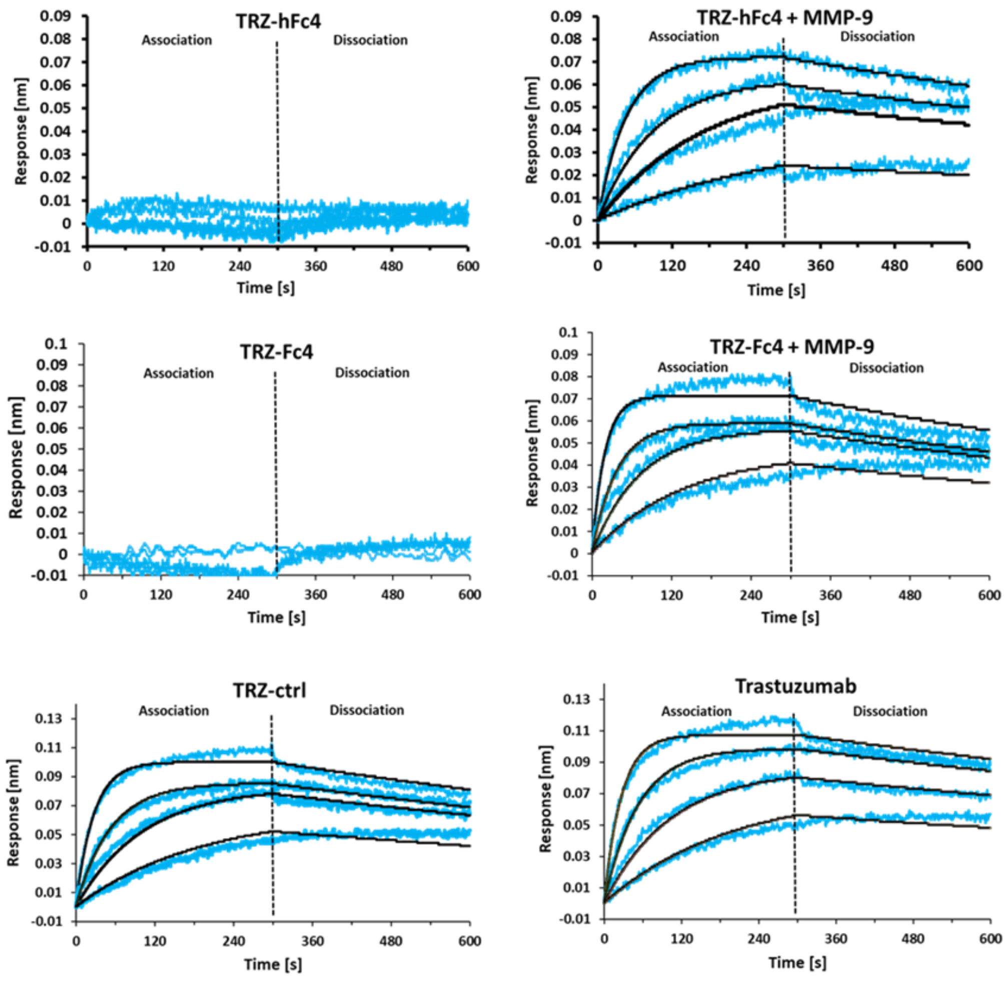

	TRZ-hFc4	TRZ-Fc4	TRZ-hFc4 +MMP-9	TRZ-Fc4 +MMP-9	Trastuzumab	TRZ-ctrl
Affinity to FcγRI [nM]	n.d.	n.d.	1.2 +/- 0.04	1.6 +/- 0.04	1.3 +/- 0.03	1.4 +/- 0.06

Figure S10. Determination of FcγRI binding kinetics of TRZ-Fc4, TRZ-hFc4 and TRZ-ctrl. In addition, TRZ-Fc4 and TRZ-hFc4 were treated with MMP-9. Trastuzumab was analyzed enabling comparison between non-cleaved and MMP-9-cleaved TRZFc4 and TRZ-hFc4. Respective antibody variants were loaded onto anti-human Fab-CH1 Biosensors (Fab2G) biosensors followed by a quenching step and subsequent association of varying concentrations of FcγRI (6.25, 12.5, 25, 50 nM). All association and dissociation steps were performed in kinetics buffer (KB). Kinetic calculations are based on at least 4 different protein concentrations and a sample without FcγRI (KB only). Errors (+/-) represent standard deviation. Data analysis was performed using ForteBio data analysis software 9.0. Binding kinetics were determined using Savitzky-Golay filtering and 1:1 Langmuir modeling.

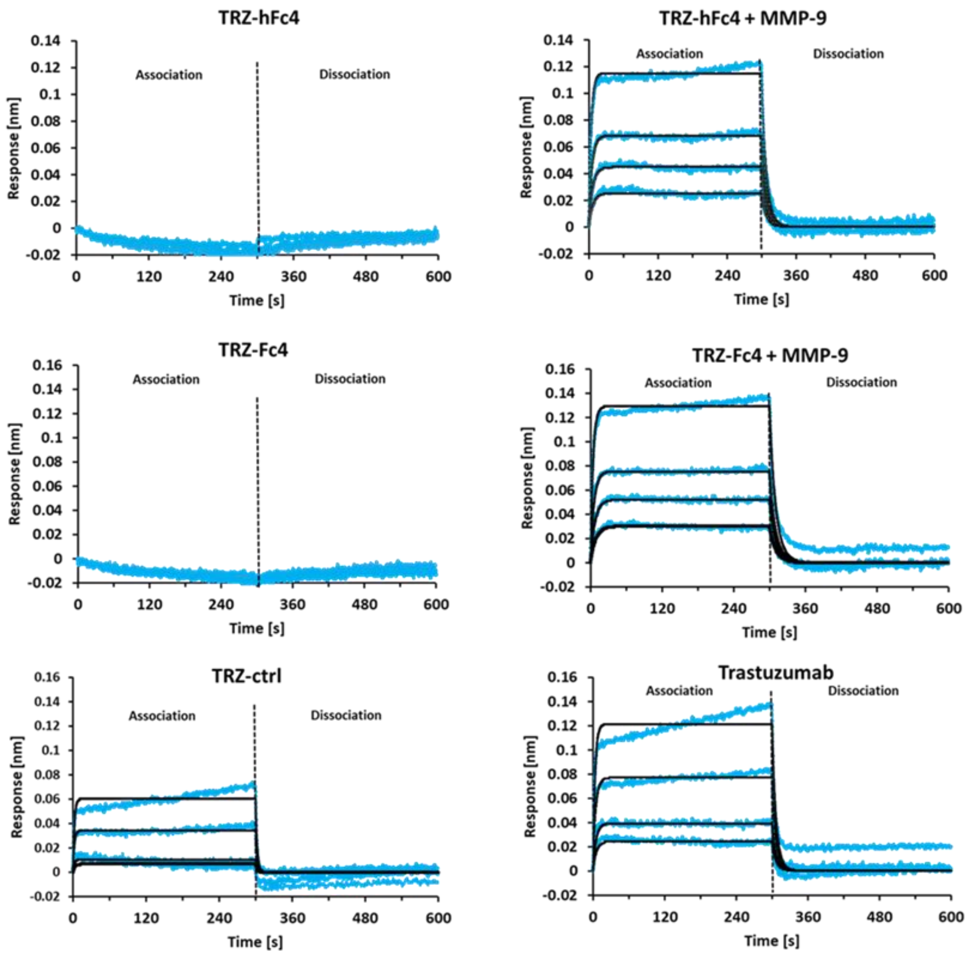

	TRZ-hFc4	TRZ-Fc4	TRZ-hFc4 +MMP-9	TRZ-Fc4 +MMP-9	Trastuzumab	TRZ-ctrl
K_D [nM] (c1q)	n.d.	n.d.	68.3 +/- 3.9	96.2 +/- 4.8	209 +/- 27.8	244 +/- 43.2

Figure S11. Determination of c1q binding kinetics of TRZ-Fc4, TRZ-hFc4 and TRZ-crtl. In addition, TRZ-Fc4 and TRZ-hFc4 were treated with MMP-9. Trastuzumab was analyzed enabling comparison between non-cleaved and MMP-9-cleaved TRZ-Fc4 and TRZ-hFc4. Respective antibody variants were loaded onto anti-human Fab-CH1 Biosensors (Fab2G) biosensors followed by a quenching step and subsequent association of varying concentrations of human c1q (12.5, 25, 50, 100 nM). All association and dissociation steps were performed in kinetics buffer (KB). Kinetic calculations are based on at least 4 different protein concentrations and a sample without c1q protein (KB only). Errors (+/-) represent standard deviation. Data analysis was performed using ForteBio data analysis software 9.0. Binding kinetics were determined using Savitzky-Golay filtering and 1:1 Langmuir modeling.

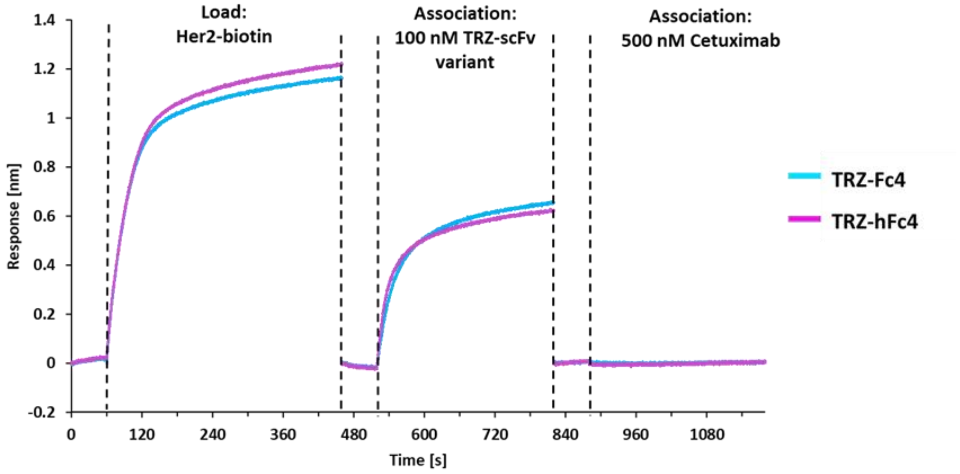

Figure S12. Epitope binning of TRZ-Fc4 and TRZ-hFc4. SAX biosensors were loaded with biotinylated Her2 (Load: Her2), followed by a quenching step. Subsequently, 100 nM TRZ-Fc4, TRZ-hFc4 was applied (Association to Her2), followed by a second association step using 500 nM cetuximab.

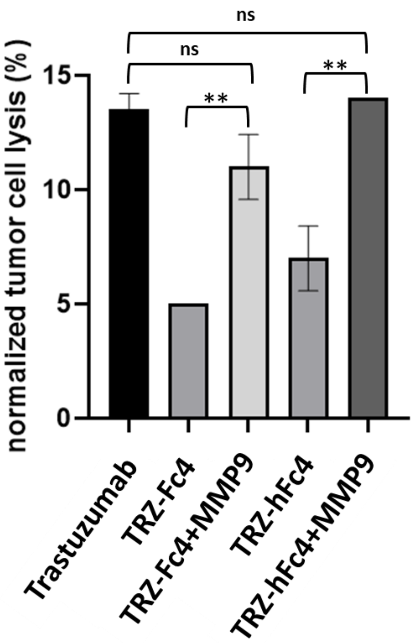

Figure S13. NK cell-mediated tumor cell killing. Cells were incubated with 100 nM of the Fc-tamed antibody variants TRZ-hFc4 or TRZ-Fc4. 100 nM Trastuzumab served as a positive control. After 17 hours tumor cell lysis was calculated. One-way ANOVA and Dunnett's post-hoc multiple comparison testing were performed for statistical analysis. ns($p>0.05$); *($p<0.05$); **($p<0.01$); ***($p<0.001$). Basal killing, depicted by dashed lines was determined using NK cells mixed with target cells, without the addition of antibody construct. Error bars represent the standard deviation derived from experimental duplicates.
